# Analysis of thymic generation of shared T‐cell receptor α repertoire associated with recognition of tumor antigens shows no preference for neoantigens over wild‐type antigens

**DOI:** 10.1002/cam4.6002

**Published:** 2023-04-28

**Authors:** Joonatan Mattila, Silja Sormunen, Nelli Heikkilä, Ilkka P. Mattila, Jari Saramäki, T. Petteri Arstila

**Affiliations:** ^1^ Research Programs Unit, Translational Immunology, Haartmaninkatu 3 (PL 21) 00014, and Medicum University of Helsinki Helsinki Finland; ^2^ Department of Computer Science Aalto University Espoo Finland; ^3^ Faculty of Medicine, Center for Vaccinology, Department of Pathology and Immunology University of Geneva Geneva Switzerland; ^4^ Department of Pediatric Cardiac and Transplantation Surgery Hospital for Children and Adolescents, Helsinki University Central Hospital Helsinki Finland

**Keywords:** cancer biology, immunology, mutations, vaccine

## Abstract

**Background:**

The number of mutations in cancer cells is an important predictor of a positive response to cancer immunotherapy. It has been suggested that the neoantigens produced by these mutations are more immunogenic than nonmutated tumor antigens, which are likely to be protected by immunological tolerance. However, the mechanisms of tolerance as regards tumor antigens are incompletely understood.

**Methods:**

Here, we have analyzed the impact of thymic negative selection on shared T‐cell receptor (TCR) repertoire associated with the recognition of either mutated or nonmutated tumor antigens by comparing previously known TCR—antigen—pairs to TCR repertoires of 21 immunologically healthy individuals.

**Results:**

Our results show that TCRα chains associated with either type of tumor antigens are readily generated in the thymus, at a frequency similar to TCRα chains associated with nonself. In the peripheral repertoire, the relative clone size of nonself‐associated chains is higher than that of the tumor antigens, but importantly, there is no difference between TCRα chains associated with mutated or nonmutated tumor antigens.

**Conclusion:**

This suggests that the tolerance mechanisms protecting nonmutated tumor antigens are non‐deletional and therefore potentially reversible. As unmutated antigens are, unlike mutations, shared by a large number of patients, they may offer advantages in designing immunological approaches to cancer treatment.

## Graphical abstract

It has been suggested that tumor neoantigens produced by mutations are more immunogenic than nonmutated tumor antigens, which are likely to be protected by immunological tolerance, although the mechanisms of tolerance are incompletely understood. Here, we show that TCRα chains associated with either type of tumor antigens are readily generated in the thymus, at a frequency similar to TCRα chains associated with nonself. This suggests that the tolerance mechanisms protecting nonmutated tumor antigens are non‐deletional and therefore potentially reversible by targeted cancer therapies.

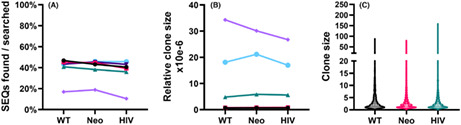

## 1 INTRODUCTION

The human immune system is capable of recognizing tumor cells, with CD8+ cytotoxic T cells as the key mediators of defense. The immune response, however, is often unable to eradicate the cancer completely, as malignant tumors have several ways of defending themselves against the immune system, whether by simply capsulating the tumor inside a dense layer of connective tissue or elaborately preventing T‐cell activation and function.^1^, ^2^, ^3^ Immunotherapies, such as enrichment and administration of tumor‐infiltrating lymphocytes, cytokine therapies, and tumor vaccines have a long history but generally modest rates of success.^4^, ^5^ More recently, checkpoint inhibitors have emerged as one of the most promising approaches to immunotherapy. By blocking pathways suppressing T‐cell function, such as CTLA‐4 or PD‐1, they prevent the tumor milieu from shutting down the T‐cell response.^5^, ^6^ Clinical trials have shown that in some patients, this induces a potent antitumor response capable of shrinking or even eradicating tumors, whereas other patients show little or no benefit.^6^, ^7^ The causes for these variable outcomes are still only partly known.

A prerequisite for successful T‐cell response against malignant cells is the presence of tumor antigens that can be recognized by the T cells. Some of these antigens are normal but dysregulated molecules, such as embryonic or lineage‐specific and overexpressed antigens.^8^ Others result from mutations and translocations which can either be part of the malignant transformation or more or less random consequences of the failure of the normal genomic control mechanisms in tumor cells. Several studies have shown that high mutational load of the tumor is an important factor predicting a positive outcome of immunotherapy.^7^, ^9^, ^10^ It is probable that the mutations provide the immune system with neoantigens (Neo) not protected by the normal immunological tolerance that should cover unmutated or wild‐type (WT) tumor antigens. Yet, targeting neoantigens, however desirable in principle, presents practical problems. The mutation profile is largely different between patients and variable even between individual tumor cells in a single patient. Therefore, specific targeting of neoantigens is likely to require mutation mapping and individualized treatment of each patient. In contrast, WT tumor antigens would provide a target shared by a significant fraction of patients, and their expression is also often important to the malignant state of the cells.^11^

Immunological tolerance is a complex phenomenon consisting of overlapping and complementary mechanisms. To what extent these different mechanisms are responsible for the apparent protection of WT tumor antigens is unclear, yet of crucial importance for understanding the potential for effective immunotherapy. More specifically, if T cells specific to WT tumor antigens are to a large extent deleted during thymic negative selection of immature autoreactive thymocytes, the effector repertoire is purged of the relevant clones and the tolerance is irreversible. If, on the other hand, WT‐specific thymocytes avoid thymic deletion in sufficient numbers, the tolerance mechanisms preventing efficient targeting of WT antigens are likely to be peripheral, such as suppressive function by regulatory T cells or lack of co‐stimulation. In contrast to thymic negative selection, such mechanisms are potentially reversible.^12^

We have recently shown that despite the high diversity of the T‐cell receptor (TCR) repertoire, a strikingly large fraction of human TCRα repertoire is shared by unrelated individuals.^13^ This shared, or public TCRα repertoire also contains numerous chains that have been linked in earlier studies to the recognition of specific antigens, both self and nonself.^14^, ^15^, ^16^ Interestingly, our data also showed that TCRα chains associated with reactivity to pancreatic islet self‐antigens are not deleted in the thymus, nor diverted to the regulatory lineage.^17^ This suggests that for some self‐antigens, at least, negative selection may not be particularly stringent. In this study, we take advantage of public TCR repertoire and previously reported TCR sequences to analyze the thymic deletion of TCR chains associated with Neo or WT tumor antigens.

## 2 MATERIALS AND METHODS

### 2.1 Databases and samples

The pediatric thymus and blood samples analyzed in this study have been previously reported,^17^, ^18^, ^19^ and the TCR sequencing data are available in the ImmuneACCESS database managed by Adaptive Biotechnologies (clients.adaptivebiotech.com/immuneaccess).^20^ Briefly, the samples included six pediatric thymus samples, obtained from immunologically healthy children undergoing corrective cardiac surgery (4–8 months old, 2/6 female). Two of the male donors were monozygotic twins. We have previously reported the twins having a genetic effect on TCR rearrangement but not on thymic selection,^18^ and the twins were analyzed as individual donors. The pediatric peripheral blood samples assessed in this study are part of the Finnish Pediatric Diabetes Register, which contains data from children with newly diagnosed type 1 diabetes.^19^ Our donors (3–14 years old, 5/10 female) belonged to the healthy sibling control cohort of the register. They had the HLA‐DR3/DR4 diabetes high‐risk genotype but no clinical symptoms during a follow‐up of 2–4 years after sampling and remained seronegative for islet antigen autoantibodies. This study was approved by the Pediatric ethics committee of Helsinki University Hospital and a written informed consent was obtained from the parents of the children. The study was done according to the principles of the Declaration of Helsinki. The details of the samples and the sequencing data have been previously published.^17^ (Table S1).

Five further samples from adult donors were obtained from the ImmuneACCESS database. Two of these samples were part of Kanakry et al.'s study assessing the origin and evolution of the reforming T‐cell repertoire after myeloablative, allogeneic bone marrow transplantation.^21^, ^22^ The samples analyzed here were obtained from two bone marrow donors (52 years old male and 61 years old female) with no reported immunological disease. Further three samples were obtained from healthy controls in a study by Delmonte et al.^23^ Although the study provided no further information on the controls, the patients' ages ranged from 19 to 56 years, and the controls were here likewise presumed to be adults.

Tumor antigen and HIV‐associated TCR sequences were obtained from the VDJdb database, and some separately collected publications.^24^, ^25^, ^26^, ^27^, ^28^ All the TCR—antigen—pairs included in this study were originally identified using HLA/peptide‐multimer recognition. The TCR—antigen—pairs included in the study are listed in Table S2.

### 2.2 Sequencing

DNA was extracted using the DNeasy or QIAsymphony kits (both from Qiagen) according to the manufacturer's instructions. The TCR genes were sequenced using the ImmunoSEQ sequencing service (Adaptive Biotech), as previously described.^29^ Briefly, ImmunoSEQ uses a standardized quantity of quality‐controlled DNA for the sequencing assay that consists of a multiplex PCR assay spanning recombined TCRα and TCRβ genes at a length that covers the entire CDR3 region and is capable of identifying both the V‐ and J‐genes for TCRα. Illumina platform was used to perform the amplicon sequencing (https://www.illumina.com/systems/sequencing‐platforms.html). The IMGT (www.imgt.org) database provided the definitions for TCRα sequences. A synthetic repertoire of TCRs and barcoded, spiked‐in synthetic templates was utilized to correct primer bias. Only in‐frame sequences were included in the analysis.

### 2.3 TCR analysis

Matching TCR sequences were searched from the databases using scripts written in Python (www.python.org), the scripts are available on request from the corresponding author. The clone sizes of the TCR sequences found were normalized by dividing the sequence counts with the total nucleated cell number estimate of the sample in question. The frequency of TCR sequences found and the clone sizes were compared using two‐tailed Wilcoxon signed rank test, with *p* < 0.05 as the limit for statistical significance. The statistical analysis was performed using the SPSS Statistics Software version 27. Unless otherwise stated, all data shown are based on amino acid sequences. The number of inserted non‐germline nucleotides was determined using online analytical tools provided by the Adaptive Biotech in its ImmunoSEQ service.

## 3 RESULTS

### 3.1 Identification of tumor antigen‐associated TCR sequences

Several recent studies have shown that a high fraction of TCRα repertoire is shared between unrelated individuals.^13^, ^30^, ^31^ These public TCRs made it possible for us to analyze the developmental fate of TCRα chains associated with the recognition of defined antigens. Three categories of specificities were analyzed: Neo and WT tumor antigens, and as a truly nonself‐control, HIV antigens since our donors were unlikely to have encountered HIV. The sequences were obtained from the previously published VDJdb database of TCRs with defined specificity and also directly from primary publications.^24^, ^25^, ^26^, ^27^, ^28^ We restricted our analysis to sequences with strict verification of specificity, with direct evidence from HLA/peptide‐multimer staining. The tumor sequences were derived from patients with several kinds of cancer and found both in the Neo‐ and the WT‐groups. All antigen‐associated TCR sequences analyzed were from CD8+ T cells. Any TCRα sequences that were previously associated with more than one of the studied antigen groups were excluded from this study to reduce bias from possible overlap between these groups. Our final reference library included 720 unique TCRα clones associated with the recognition of WT, Neo, or HIV epitopes (WT: *N* = 201, Neo: *N* = 366, HIV: *N* = 153; Table S2). Interindividual sharing of the TCRβ repertoire is much less common than that of TCRα, and our search for matches between previously defined tumor‐associated TCRβ sequences and our own samples produced few hits. TCRβ repertoire was therefore not analyzed further.

### 3.2 Generation of tumor antigen‐associated TCRα chains in the thymus

To analyze how TCRα sequences associated with the three groups of antigens are generated in the thymus, we first compared our database of TCRα— antigen—pairs with our in‐frame sequence libraries from thymus samples obtained from six immunologically healthy children (Table S1). In all the studied groups, a substantial number of matching sequences identical in both V gene and CDR3 sequences were found. There was no difference between WT and Neo‐associated sequences as the average fraction of matching sequences was 79.2/201 (39.4%) and 144/366 (39.3%), respectively (ns). Sequences associated with HIV were found at a roughly similar frequency (54.8/153 = 35.8%) as well (ns when compared with WT; Table S3). When found in one of our samples, the sequences were mostly found in others, as well, with no difference between the different kinds of antigens. On the average, the identified WT, Neo, or HIV‐associated TCRα sequences were found in 3.8, 3.7, and 3.7 of the six thymus samples, respectively (Table S4).

**FIGURE 1:**
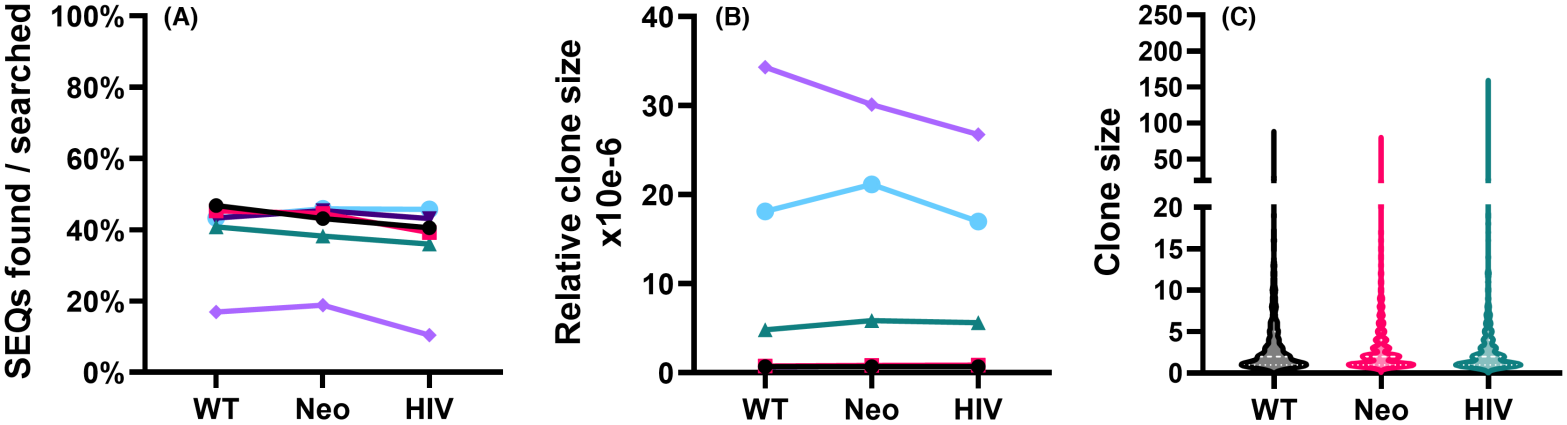
Tumor antigen and HIV‐associated T‐cell receptor (TCR)α sequences in thymus samples. (A) The fraction of TCRα sequences found in the samples. (B) The average relative clone size of the TCRα sequences found in the samples, defined as sequence recurrence divided by the estimated number of nucleated cells in the sample. (C) The distribution of clone sizes of the TCRα sequences found in the samples. For (A) and (B), each symbol represents an individual sample and values from the same sample are connected by lines. In (C), the *y*‐axis represents the range of the clone sizes, and the width of each plot shows the relative distribution of the clone sizes. The bolded dashed lines indicate the median of the clone sizes, and the dotted lines represent the borders of the outer quartiles of the clone sizes. Note the discontinuous *y*‐axis.

Because the TCR sequencing method used by Adaptive Biotechnologies is based on genomic DNA, it allows us to reasonably estimate the relative frequency of the cells expressing the TCRα chains analyzed within the samples. To normalize these frequencies in samples of different size, we calculated the average relative clone size of the TCRα clones found in the samples, defined as sequence recurrence divided by the estimated number of nucleated cells in the sample. The two types of tumor‐associated sequences had very similar relative clone sizes on average (WT = 9.9 × 10^−6^, Neo = 9.9 × 10^−6^, ns), again with only a small difference between them and the HIV‐associated sequences (HIV = 8.6 × 10^−6^, ns when compared with WT). The distribution of the clone sizes was likewise similar (Figure 1; Figure S1).

### 3.3 Persistence of tumor antigen‐associated TCRα chains in the peripheral repertoire

As our thymocyte samples were unsorted and thus contained immature thymocytes, a substantial fraction of the TCRα sequences detected might still get eliminated from the repertoire in the thymic selection processes and never reach periphery.^32^ We next analyzed a set of 10 pediatric blood samples, from children 3 to 14 years old (Table S1). The details of these samples have been previously published.^17^ Sequences associated with WT and Neo tumor antigens were detected at a similar frequency in the blood samples (34.2/201 = 17.0% and 64.7/366 = 17.7%, respectively, ns), and this frequency was also similar in HIV‐associated sequences (25.6/153 = 16.7%, ns). In all three groups of antigens, the fraction of sequences found in the periphery was smaller than in thymus, most likely because the blood samples were smaller. Each of the found TCRα sequences was on average found in 3.8 of the 10 samples analyzed in every group studied (Table S4).

**FIGURE 2:**
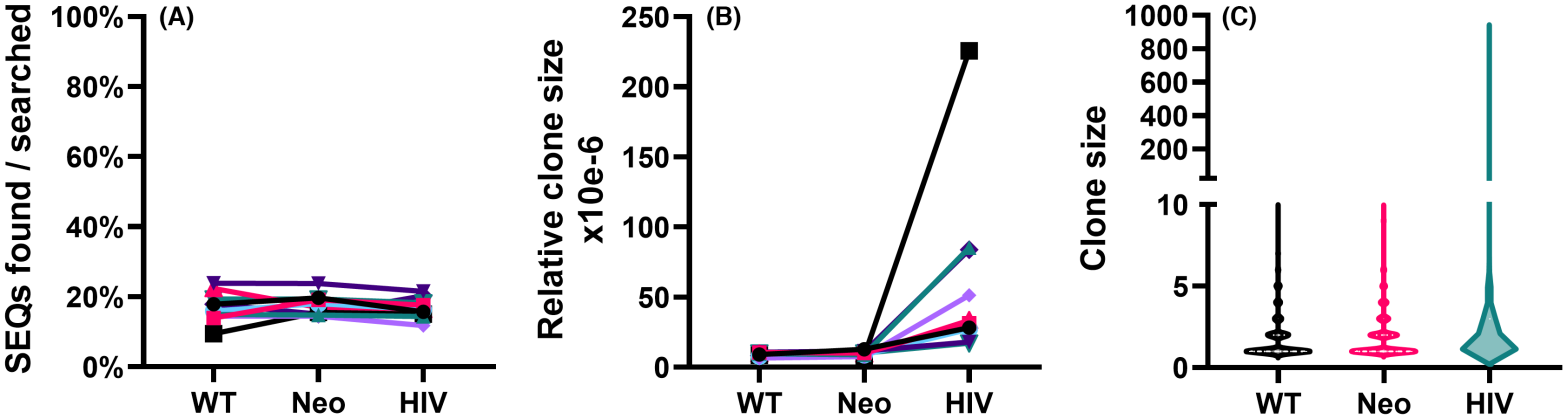
Tumor antigen and HIV‐associated T cell receptor (TCR) α sequences in pediatric peripheral blood samples. (A) The fraction of TCRα sequences found in the samples. (B) The average relative clone size of the TCRα sequences found in the samples, defined as sequence recurrence divided by the estimated number of nucleated cells in the sample. (C) The distribution of clone sizes of the TCRα sequences found in the samples. For (A) and (B), each symbol represents an individual sample and values from the same sample are connected by lines. In (C), the *y*‐axis represents the range of the clone sizes, and the width of each plot shows the relative distribution of the clone sizes. The bolded dashed lines indicate the median of the clone sizes, and the dotted lines represent the borders of the outer quartiles of the clone sizes. Note the discontinuous *y*‐axis.

The average relative clone sizes of the identified tumor TCRα sequences in the pediatric blood samples were like those in the thymus. Again, there was no difference between the two types of tumor antigens, with average relative clone sizes of 9.1 × 10^−6^ and 9.9 × 10^−6^ for the WT and the Neo tumor antigen‐associated sequences, respectively (ns). However, in the periphery, the average relative clone size of HIV‐associated sequences (6.1 × 10^−5^) was significantly higher than that of the tumor‐associated sequences (*p* < 0.01). Unlike in the thymus, the peripheral HIV‐associated clones also contained a subset found at a clearly higher frequency compared to the majority of clones (Figure 2; Figure S2).

### 3.4 Convergent recombination in tumor antigen‐associated TCRα sequences

**FIGURE 3:**
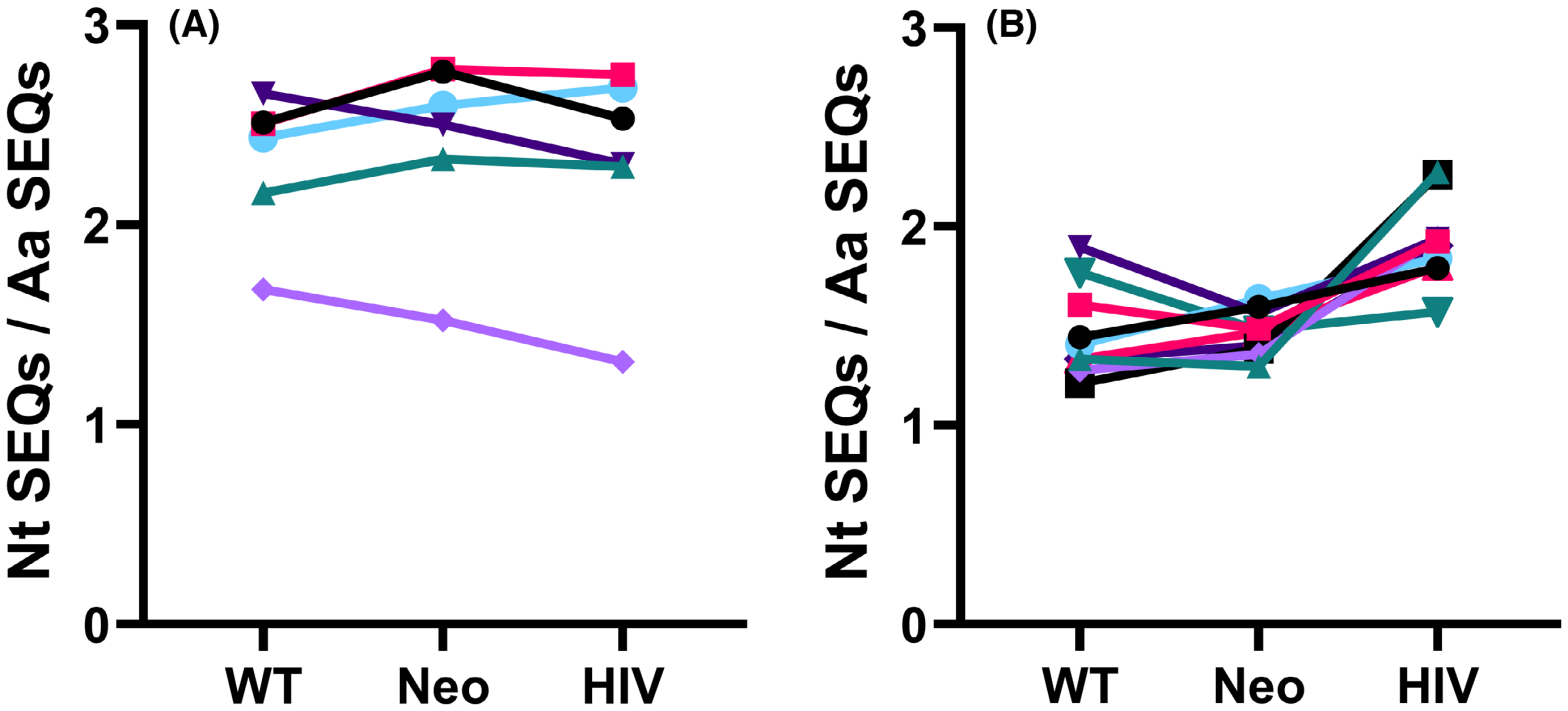
Convergent recombination of tumor antigen and HIV associated T cell receptor (TCR) α amino acid sequences. (A) Thymus samples and (B) pediatric blood samples. The average number of nucleotide sequences encoding each identified amino acid sequence is shown. Each symbol represents an individual sample and values from the same sample are connected by lines.

We then analyzed the nucleotide sequences encoding the amino acid sequences associated with the WT, Neo, and HIV‐associated antigens but found no significant difference between them in the thymus. On average, each identified amino acid sequence was encoded by the same number of different nucleotide sequences (WT 2.3, Neo 2.4, HIV 2.3, ns). In the blood samples, the tumor antigen‐associated amino acid sequences were encoded on average by 1.46 (WT) and 1.47 (Neo) nucleotide chains (ns). The average number of nucleotide sequences encoding each HIV‐associated amino acid sequence was significantly higher than that of either of the tumor antigen‐associated groups (1.92, *p* < 0.01 vs Neo, *p* < 0.01 vs WT; Figure 3).

### 3.5 Tumor antigen‐associated TCRα chains in adult peripheral blood samples

To see whether the repertoire associated with tumor antigens undergoes changes with age, we next searched for peripheral blood samples from adult donors. For inclusion, the following criteria had to be fulfilled: The samples were obtained from donors with no reported immunological disease, the samples included unselected PBMC, and the sequencing was done with the ImmunoSEQ assay, using the same methodology as ours. Five adult samples matching these criteria were available. Two of these samples were thoroughly described in the original publication, one male and one female, 52 and 61 years old, respectively.^21^, ^22^ The remaining three were healthy controls in a study focusing on four unrelated 19–56‐year‐old patients with SASH3 deleterious variants.^23^ Although these samples were not described further in the original publication, we have no reason to believe that the donor age and sex would significantly differ from the patients.

**FIGURE 4:**
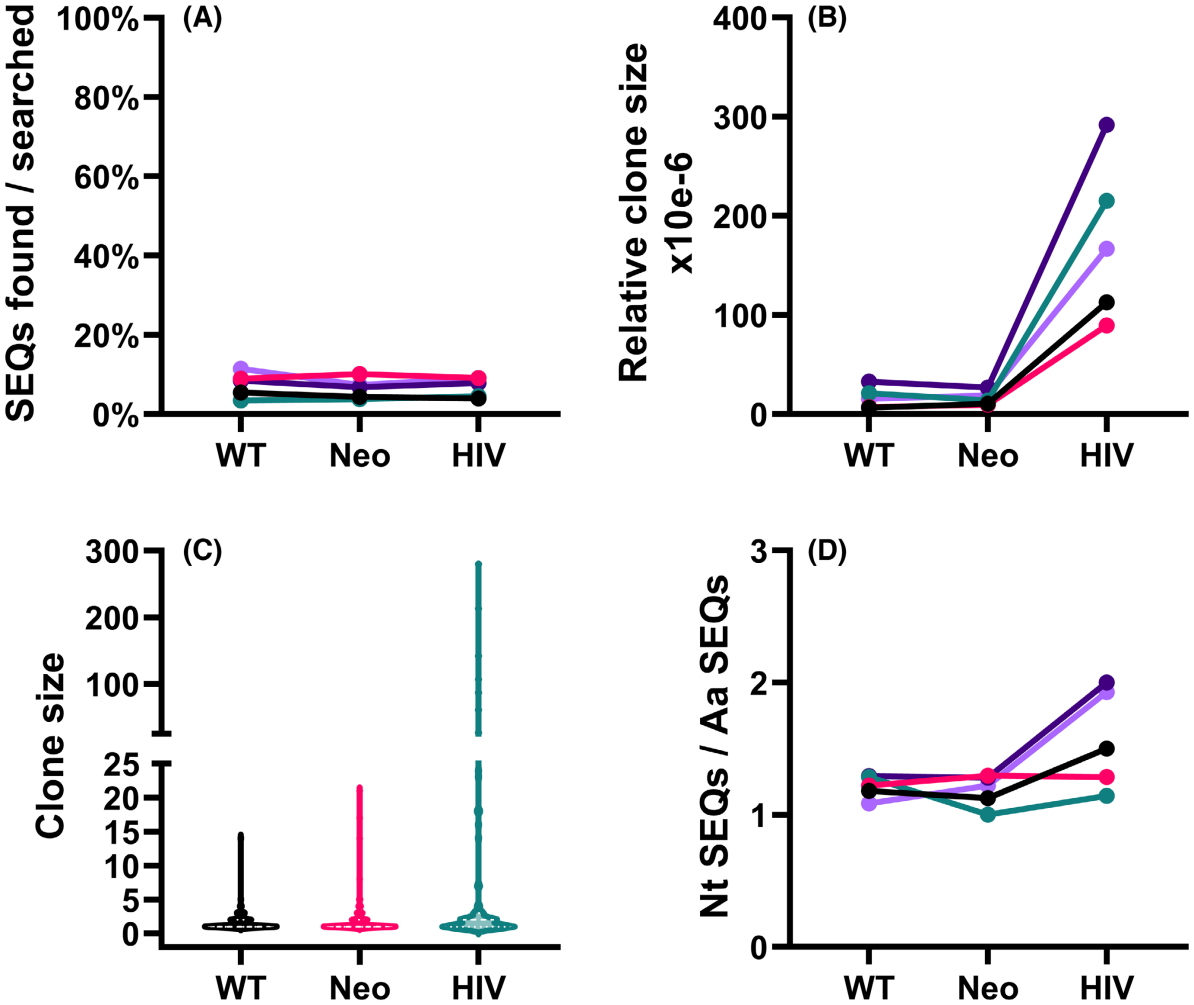
The appearance, relative clone sizes and nucleotide composition of tumor antigen and HIV‐associated T cell receptor (TCR) α sequences in adult peripheral blood. (A) The fraction of TCRα sequences found in the samples. (B) The average relative clone size of the TCRα sequences found in the samples, defined as sequence recurrence divided by the estimated number of nucleated cells in the sample. (C) The distribution of clone sizes of the TCRα sequences found in the samples. (D) The average number of nucleotide sequences encoding each identified amino acid sequence is shown. For (A), (B), and (D), each symbol represents an individual sample and values from the same sample are connected by lines. In (C), the *y*‐axis represents the range of the clone sizes, and the width of each plot shows the relative distribution of the clone sizes. The bolded dashed lines indicate the median of the clone sizes, and the dotted lines represent the borders of the outer quartiles of the clone sizes. Note the discontinuous *y*‐axis.

We found numerous exact CDR3, and V‐gene matches between these blood samples and our TCRα—antigen—pair library in both the WT and the Neo tumor antigen‐associated TCRα groups, as well as in the HIV‐associated control group. These TCRα chains with known antigen association were found at similar frequencies across all the groups (WT = 15.2/201 = 7.6%, Neo = 23.8/366 = 6.5%, HIV = 10.6/153 = 6.9%, ns). Each of these TCRα sequences was on the average found in 1.6 of the five samples analyzed, for every type of antigen (Table S4). There was also no significant difference in the average relative clone size or the number of nucleotide sequences used to encode the amino acid sequences between either of the tumor antigen types or the HIV‐associated sequences (Figure 4). The distribution of clone sizes of the tumor antigen and HIV‐associated TCR α sequences in each individual adult peripheral blood sample analyzed can be found in Figure S3.

### 3.6 Analysis of non‐germline insertions in tumor antigen‐associated TCRα sequences

It has been previously reported that the public TCRα clones have in general fewer inserts than non‐shared TCRα clones.^13^ Consistent with this, in the thymus, a large fraction of tumor antigen‐associated sequences had a germline nucleotide sequence (WT 44% vs Neo 47%, ns), while in the HIV‐associated sequences the fraction was even higher (57%, *p* < 0.05). Correspondingly, the average number of inserts was small in all the public sequences analyzed (1.1 in both WT and Neo, ns.), with the smallest numbers appearing in the HIV‐associated sequences (0.8, *p* < 0.05 when compared with WT; Figure S4).

In the periphery, the results were on the average similar, with 48% of WT and 57% of Neo‐associated sequences in the germline configuration (ns), compared with 64% of HIV‐associated sequences (*p* < 0.05 when compared with WT). The average number of inserts was likewise similar between the tumor antigen‐associated sequences (WT 0.7 vs Neo 0.9, ns) and not significantly different from that in HIV‐associated sequences (0.6). However, a closer analysis of individual blood samples showed that unlike the WT‐associated sequences, the Neo‐associated sequences contained a small fraction of sequences with a clearly higher insert number, observable in seven out of 10 samples (Figure S5).

**FIGURE 5:**
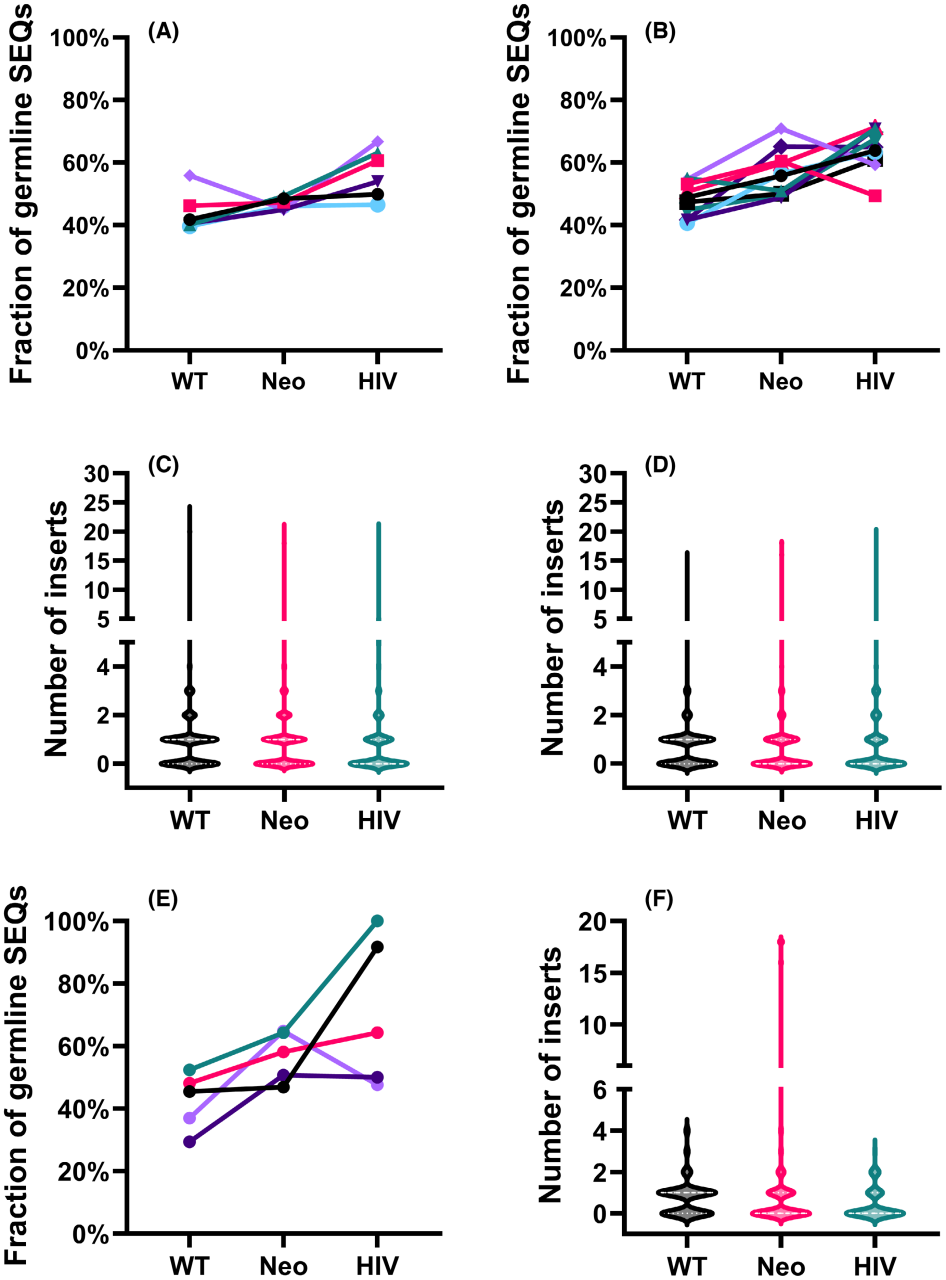
Analysis of non‐germline nucleotides in tumor antigen and HIV‐associated T cell receptor (TCR) α amino acid sequences. (A) The fraction of germline sequences in thymus and (B) pediatric blood samples. (C) The number of non‐germline nucleotides, shown as the distribution in all thymus samples and (D) pediatric blood samples combined. (E) The fraction of germline sequences in adult samples. (F) The number of non‐germline nucleotides, shown as the distribution in adult samples. For (A), (B), and (E), each symbol represents an individual sample and values from the same sample are connected by lines. In (C), (D), and (F), the *y*‐axis represents the range of the insert number, and the width of each plot shows the relative distribution of the insert number. The bolded dashed lines indicate the median, and the dotted lines represent the borders of the outer quartiles of the insert numbers. Note the discontinuous *y*‐axis.

In the adult peripheral blood samples, the TCRα nucleotide sequences associated with the cancer groups were also equally close to the germline on average (WT = 42%, Neo = 57%, ns), whereas the fraction of TCRα sequences in germline configuration was slightly but again not significantly larger in the HIV control group (HIV = 71%). The Neo‐associated TCRα sequences had on average a slightly higher number of inserts than the WT‐associated sequences (WT = 0.75, Neo = 1.44), although this difference was not significant. The HIV‐associated group had on average the smallest number of inserts (HIV = 0.38, ns; Figure 5). Again, a closer analysis of individual blood samples showed a small separate group of Neo‐associated sequences with a higher insert number, observable in four out of five samples (Figure S6).

## 4 DISCUSSION

In recent years, cancer immunotherapies have emerged as novel, specific options of cancer treatment and some of these immune defense enhancing treatments show considerable promise, especially when used in combinations with other cancer treatments.^33^, ^34^ The clinical outcomes, however, have been highly variable both between different types of cancer and even between different individuals with the same type of cancer.^33^, ^35^, ^36^ Although the tumor microenvironment and the general suppression of the host immune system have both been shown to contribute to this variability, a major factor is the mutation profile of the cancer.^7^ Generally, the more neoantigens a cancer contains or the higher the tumor mutation burden, defined as the number of non‐synonymous single nucleotide variants, the better the outcome of a checkpoint inhibitor treatment.^7^, ^37^ One simple explanation could be that, since in the anti‐immunogenic tumor microenvironment, only 2–5 neoantigens per the potentially hundreds of available neoantigens are usually able to raise a proper T‐cell response, the sheer increase in number of neoantigens would provide more potential targets for the immune system and increase the chance of a potent response.^7^ A major factor suggested to favor the recognition of neoantigens is that T cells potentially recognizing them have not been eliminated during the thymic negative selection, which may not be the case for T cells specific to WT tumor antigens.^7^, ^38^, ^39^ However, some people with tumors of low mutation burden still respond to the immune checkpoint inhibitor treatments relatively well, whereas others with tumors of high mutation burden do not,^9^, ^40^, ^41^ which suggests that the final picture is more complicated. A recent study using a transgenic mouse model to track the thymic generation and selection of T‐cell repertoire suggests that the generation of self‐tolerance is not a clear‐cut binary selection process which would rely on the characteristics of a single TCR sequence. Rather, it is the outcome of a relatively weak thymic selective pressure combined with probabilistic quorum decision‐making, emphasizing the importance of the collective pool of T cells.^42^

It is important to note that targeting WT tumor antigens may in some situations be desirable. First, the neoantigen profile of each cancer patient is unique by definition and thus specifically targeting neoantigens requires individual tumor exome or genome sequencing prior to treatment.^39^ Indeed, specific neoantigen targeting therapies would in most cases have to be individually designed for each patient, whereas with WT tumor antigens, a single drug could suit multiple patients. Besides individual patients, the mutation profile is usually different in even individual tumor cells within one patient, increasing the possibility of immune evasion. Targeting antigens shared by most or all tumor cells, such as WT lineage markers, may decrease this risk, especially if the selected antigens are crucial in the tumorigenic process. It should also be noted that even though targeting neoantigens may be easier, some tumors are by nature poor in mutations, and in such cases targeting WT tumor antigens might be a better, or even the only plausible strategy for antigen‐specific immunotherapy.^43^

In this study, we found no significant difference between WT and Neo‐associated TCRα sequences in the fraction of sequences found in the thymus or in their respective relative clone sizes. Both were also comparable to sequences associated with nonself HIV sequences. Overall, the clonal distributions in the thymus were similar in each group, probably because they mostly reflect the recombination events, which are not affected by specificity. However, in periphery, the average relative clone size of HIV‐associated sequences was over six times bigger than the average relative clone size of tumor antigen‐associated sequences, an indication that thymic selection had a distinct impact on self versus nonself sequences, such that a higher fraction of nonself‐associated chains survived. Importantly, there was again no difference between WT and Neo‐associated sequences. Similar findings were also obtained in the analysis of adult peripheral blood samples. In the thymus, sequences associated with the two tumor antigen types were also similar in the fraction of germline CDR3s and the average number of inserts. Intriguingly, in the periphery, the Neo‐associated sequences contained a small subset with relatively high number of inserts, not seen in the WT‐associated sequences. This may suggest slight qualitative differences in the pool of precursors from which the antitumor responses are recruited.

The main shortcoming of our study is the fact that it only addresses one of the chains of a heterodimer, and there is little reason to expect that the TCRα chains we identify would share identical TCRβ partners in different individuals. Indeed, studies of paired TCRs have shown that one of the chains is often public, but TCRs with both chains being shared are rare.^44^ So far, the methods to analyze paired TCRs are limited to some thousands of cells per sample, so the clonal frequency of the TCRα chains studied here, at 1 × 10^−5^, is far below the threshold of detection.^44^, ^45^ It is therefore currently impossible to analyze preimmune TCR repertoire on a heterodimer level, and further studies will be necessary to establish the true promiscuity of TCR pairing and antigen recognition.

However, several studies have shown that a public TCRα chain, paired with a variety of different TCRβ chains, can retain its specificity, while the affinity to the antigen changes.^46^, ^47^, ^48^, ^49^, ^50^ For example, Liang et al showed that a HER2369 peptide‐specific TCRα was able to recognize its specific antigen in combination with four different TCRβ chains associated with specificity not related to HER2.^47^ Moreover, Yokosuka et al. showed that the pool of potentially suitable TCRβ partners can be very large.^48^ In their study, a HIV gp160‐specific TCRα chain retained its specificity with one third of randomly selected TCRβ partners. Indeed, others have suggested that TCRs dominated by one chain are common in the TCR repertoire, and in one study up to 20% of antigen‐specific memory T cells were shown to use an α‐chain‐centric TCR.^49^

In a direct demonstration that single chain data can predict specificity, we have previously shown that the human thymus is inefficient in deleting islet antigen‐specific TCRα chains,^17^ and Linsley et al. have further shown that such public TCRα chains are indeed enriched among human islet antigen‐reactive CD4+ memory T cells.^51^ These cells have identical TCRα sequences paired with different TCRβ sequences and seem to have a role in the pathogenesis of autoimmune type 1 diabetes, suggesting that shared TCRα chains, even with unknown TCRβ partners, may have functional relevance. Linsley et al. have suggested that while TCRα sequences might have a dominant role in antigen recognition, they might also have a higher potential for self‐recognition via shorter, more germline‐like sequences than their TCRβ counterparts.

## 5 CONCLUSIONS

In conclusion, we show that the thymus generates TCRα chains associated with the recognition of both WT and Neo tumor antigens at equal frequencies and their average relative clone size is also equal. When peripheral T cells were analyzed, the WT and Neo‐associated TCRα repertoires were likewise similar, but now differed from the nonself HIV‐associated TCRα chains. In other words, our analysis suggests that thymic‐negative selection has a similar impact on both WT and Neo‐associated TCRα repertoires. So, if not thymic‐negative selection, what protects WT tumor antigens since clinical observations clearly point out neoantigens as easier targets? Self‐antigens are also protected by peripheral tolerance, including regulatory T cells and other immunosuppressive mechanisms, and these may also be less efficient in the case of neoantigens. Importantly, most of these peripheral mechanisms are, at least in theory, reversible, with a suitably tailored immunotherapy. Of special interest is the possibility of combining checkpoint inhibitors with antigen‐specific immunostimulatory treatments, such as tumor vaccines or other approaches targeting shared, preferably functionally important tumor antigens, which may often be WT.^52^

## AUTHOR CONTRIBUTIONS

**Joonatan Mattila:** Conceptualization (equal); formal analysis (lead); funding acquisition (supporting); investigation (equal); software (equal); visualization (lead); writing – review and editing (equal). **Silja Sormunen:** Formal analysis (equal); software (equal). **Nelli Heikkilä:** Conceptualization (equal); formal analysis (equal); investigation (equal); software (equal). **Ilkka P Mattila:** Investigation (equal); resources (equal). **Jari Saramäki:** Formal analysis (equal); software (equal). **T Petteri Arstila:** Conceptualization (equal); funding acquisition (lead); investigation (equal); project administration (equal); resources (equal); writing – review and editing (equal).

## FUNDING INFORMATION

This study was funded by impartial foundations and research programs; we did not receive any corporate funding. Grants from Finnish Medical Foundation (grant no. 4115), Foundation for Pediatric Research, and the MD PhD program of the Faculty of Medicine of the University of Helsinki were used to pay for human resources. The grants from Waldemar von Frenckell Foundation, Liv och Hälsa Foundation, and Foundation for Pediatric Research facilitated material and sequencing costs for the study.

## CONFLICT OF INTEREST STATEMENT

The authors have no conflict of interest.

## Supporting information


Figure S1.

Figure S2.

Figure S3.

Figure S4.

Figure S5.

Figure S6.

Table S1.

Table S2.

Table S3.

Table S4.

## Data Availability

All TCRα sequences analyzed are available at ImmuneACCESS database maintained by Adaptive Biotechnologies (clients.adaptivebiotech.com/immuneaccess). All TCRα—antigen—pairs analyzed can be found in Table S2. All Python (www.python.org) scripts are available at request from joonatan.mattila@helsinki.fi.
