# Supplementary figures and images for: Analysis of thymic generation of shared T‐cell receptor α repertoire associated with recognition of tumor antigens shows no preference for neoantigens over wild‐type antigens
Source: Cancer Med. 2023 Apr 28;12(12):13486–96. doi: 10.1002/cam4.6002 (PMC10315763; doi:10.1002/cam4.6002)

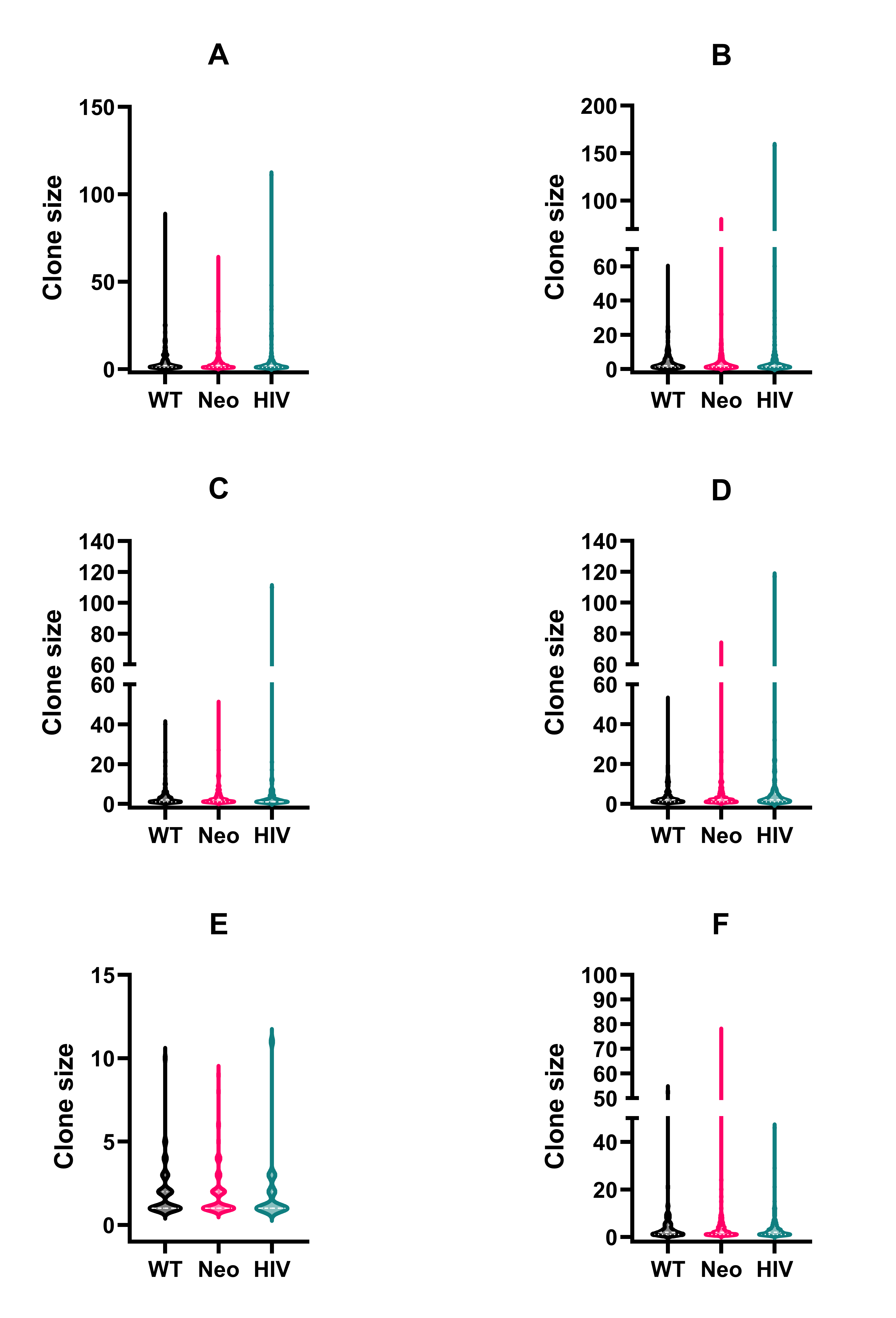

Supplement: Supplementary file 1 — Figure S1. [file CAM4-12-13486-s007.tif]

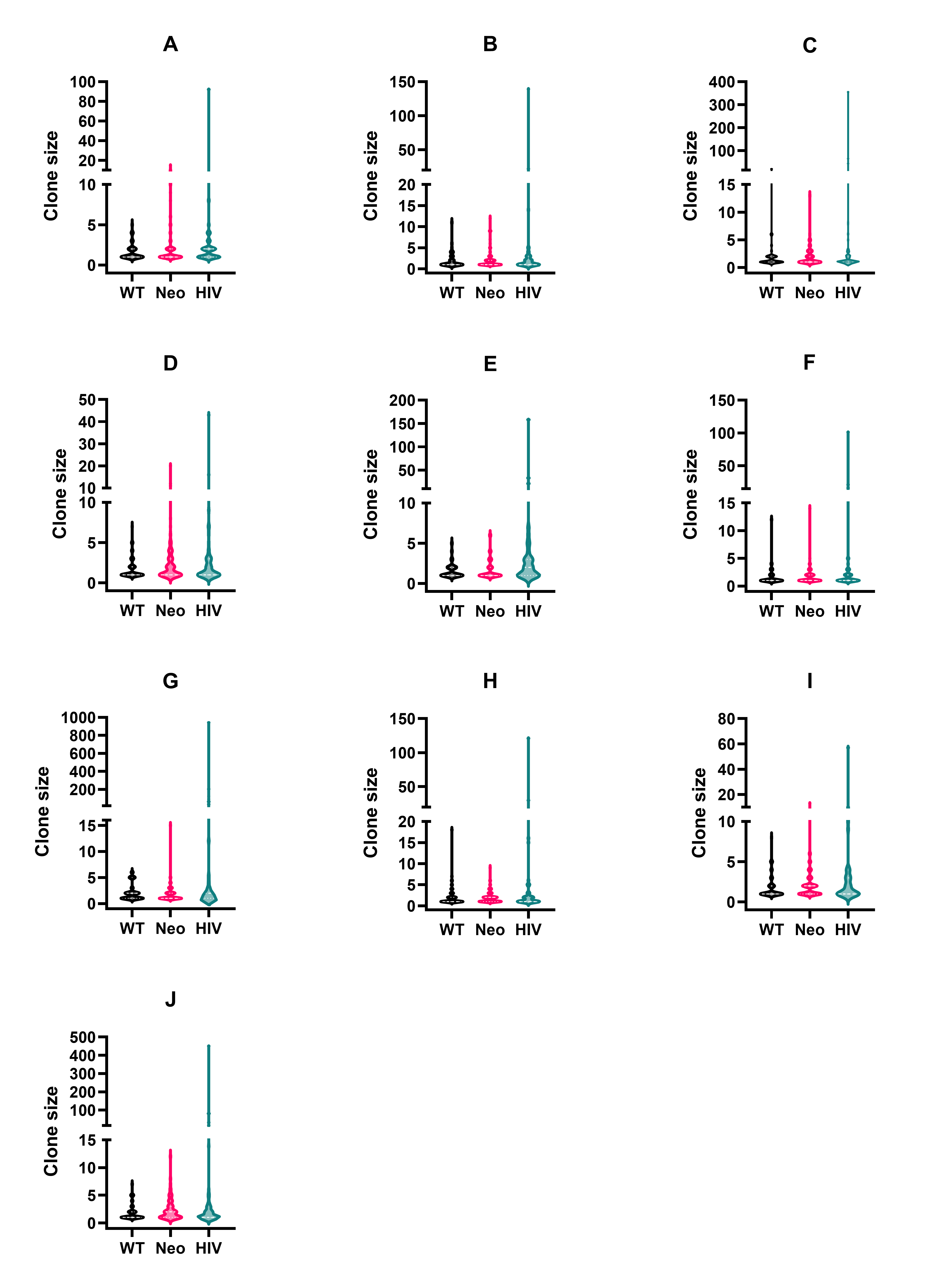

Supplement: Supplementary file 2 — Figure S2. [file CAM4-12-13486-s005.tif]

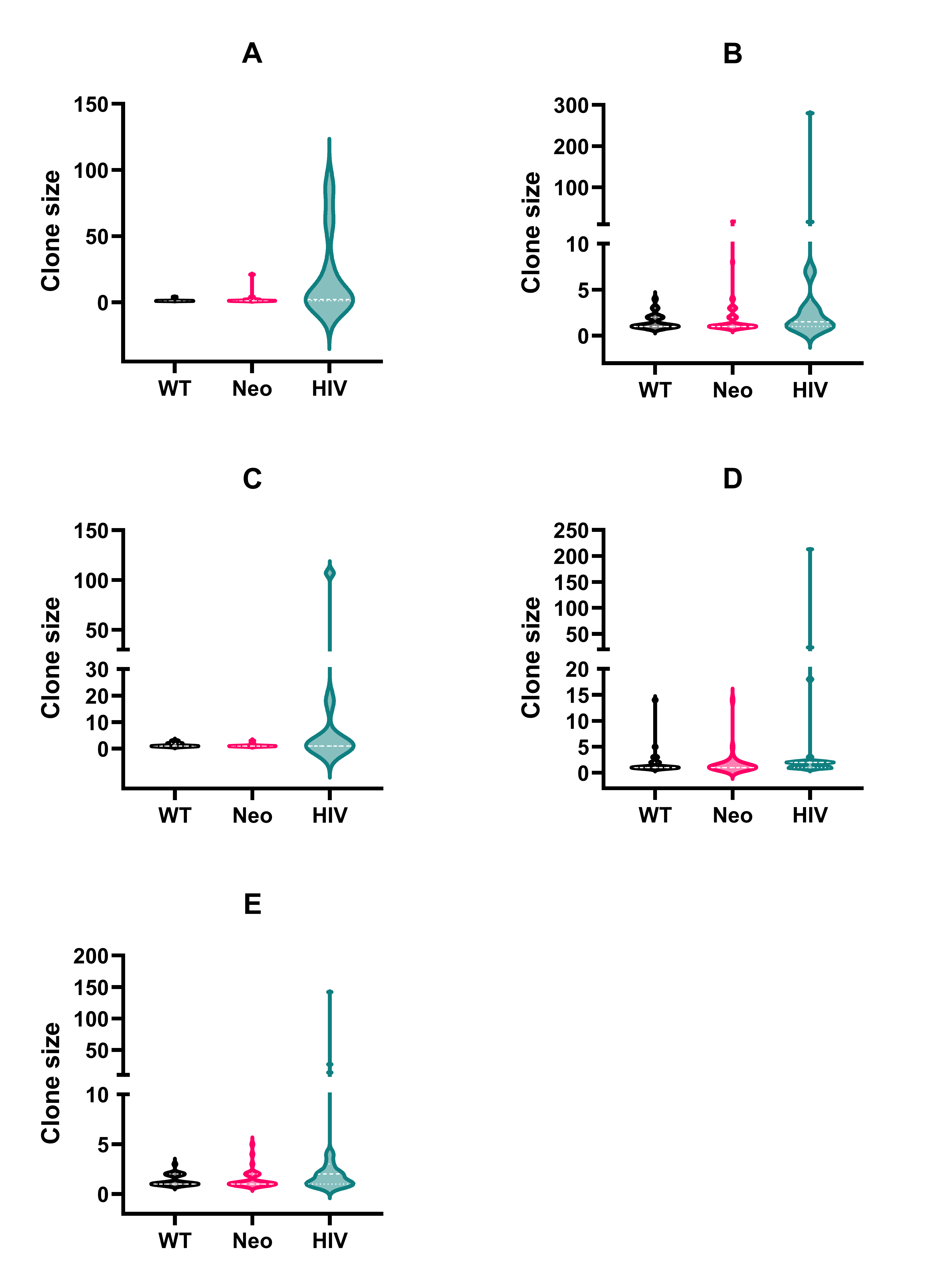

Supplement: Supplementary file 3 — Figure S3. [file CAM4-12-13486-s004.tif]

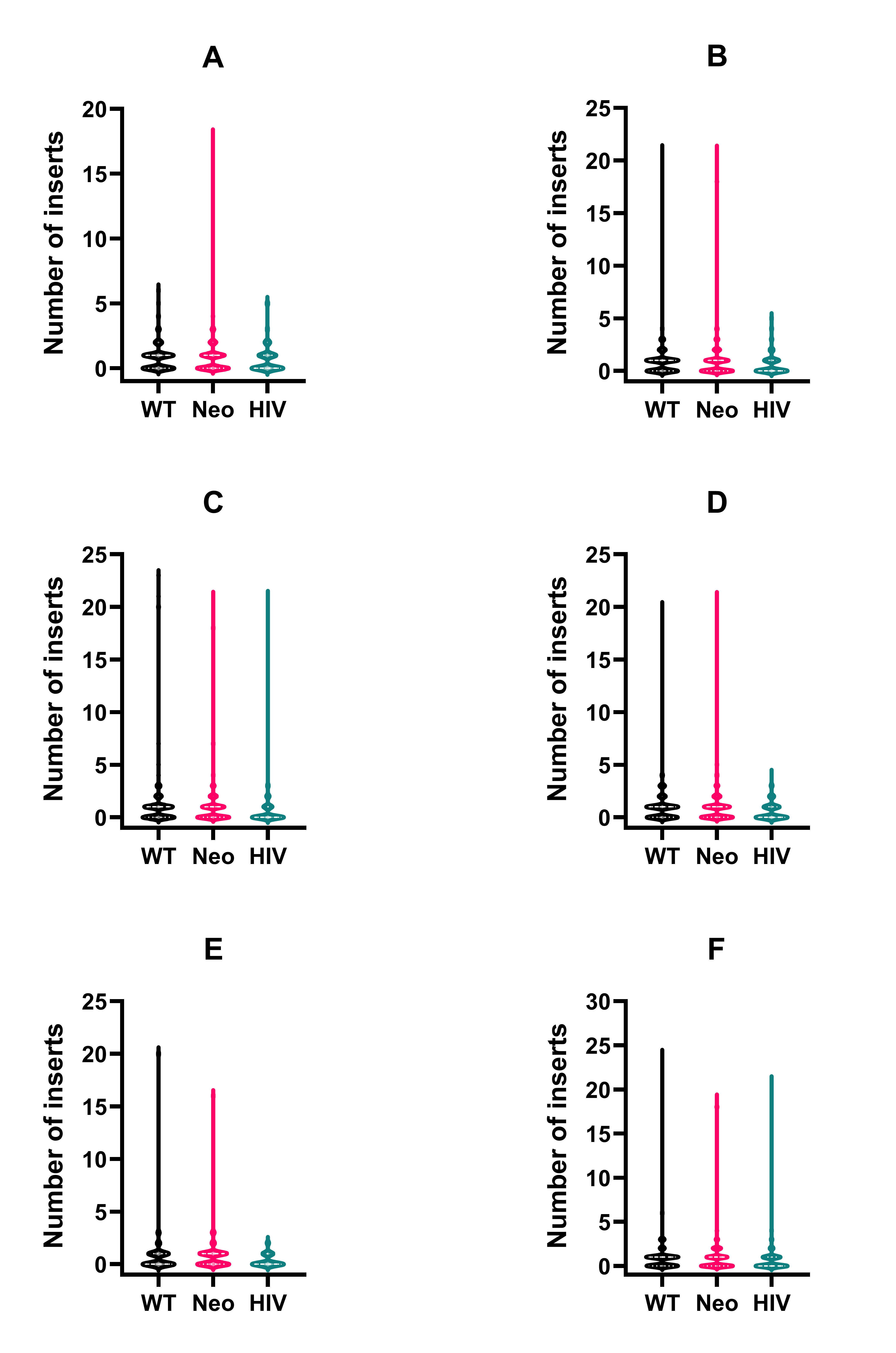

Supplement: Supplementary file 4 — Figure S4. [file CAM4-12-13486-s010.tif]

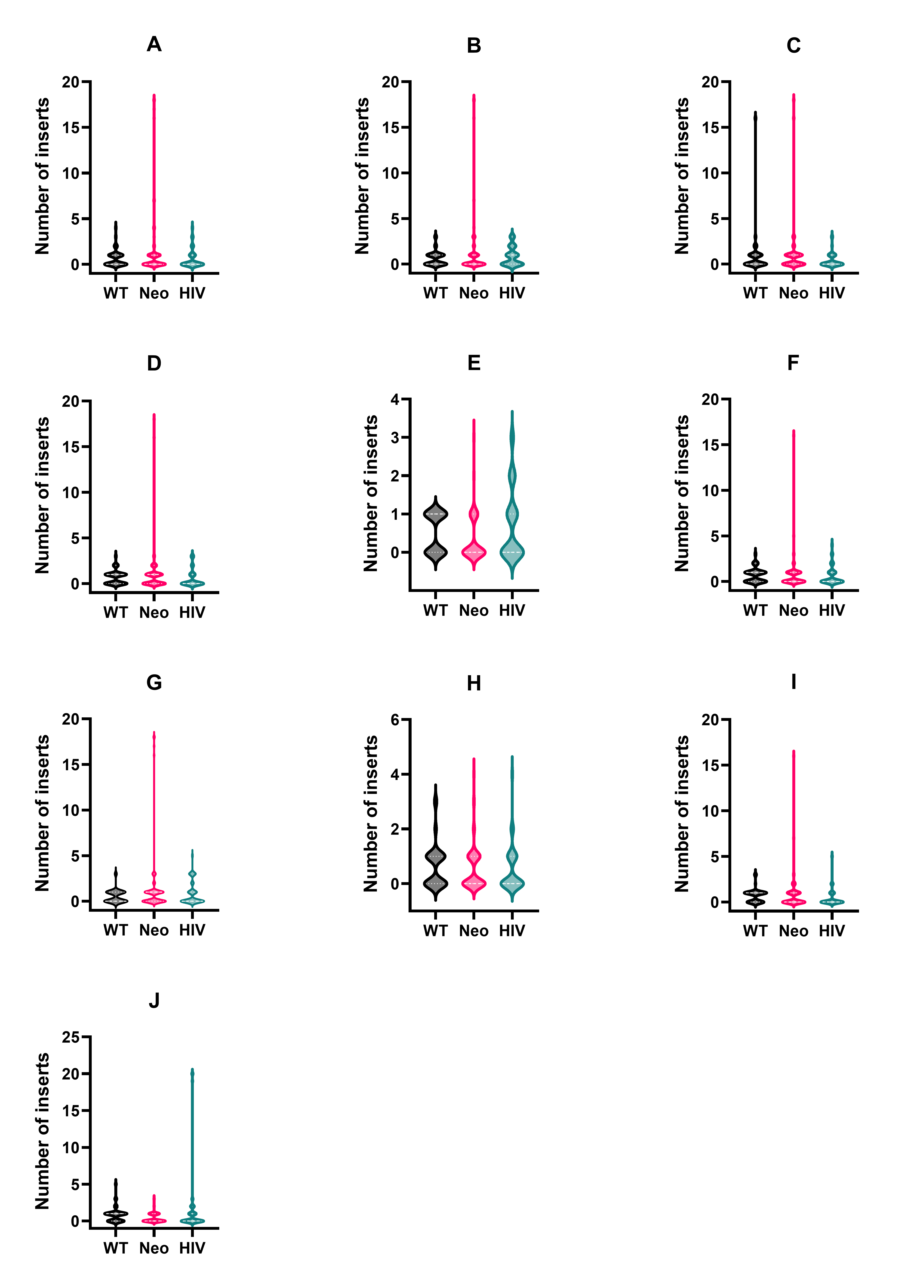

Supplement: Supplementary file 5 — Figure S5. [file CAM4-12-13486-s001.tif]

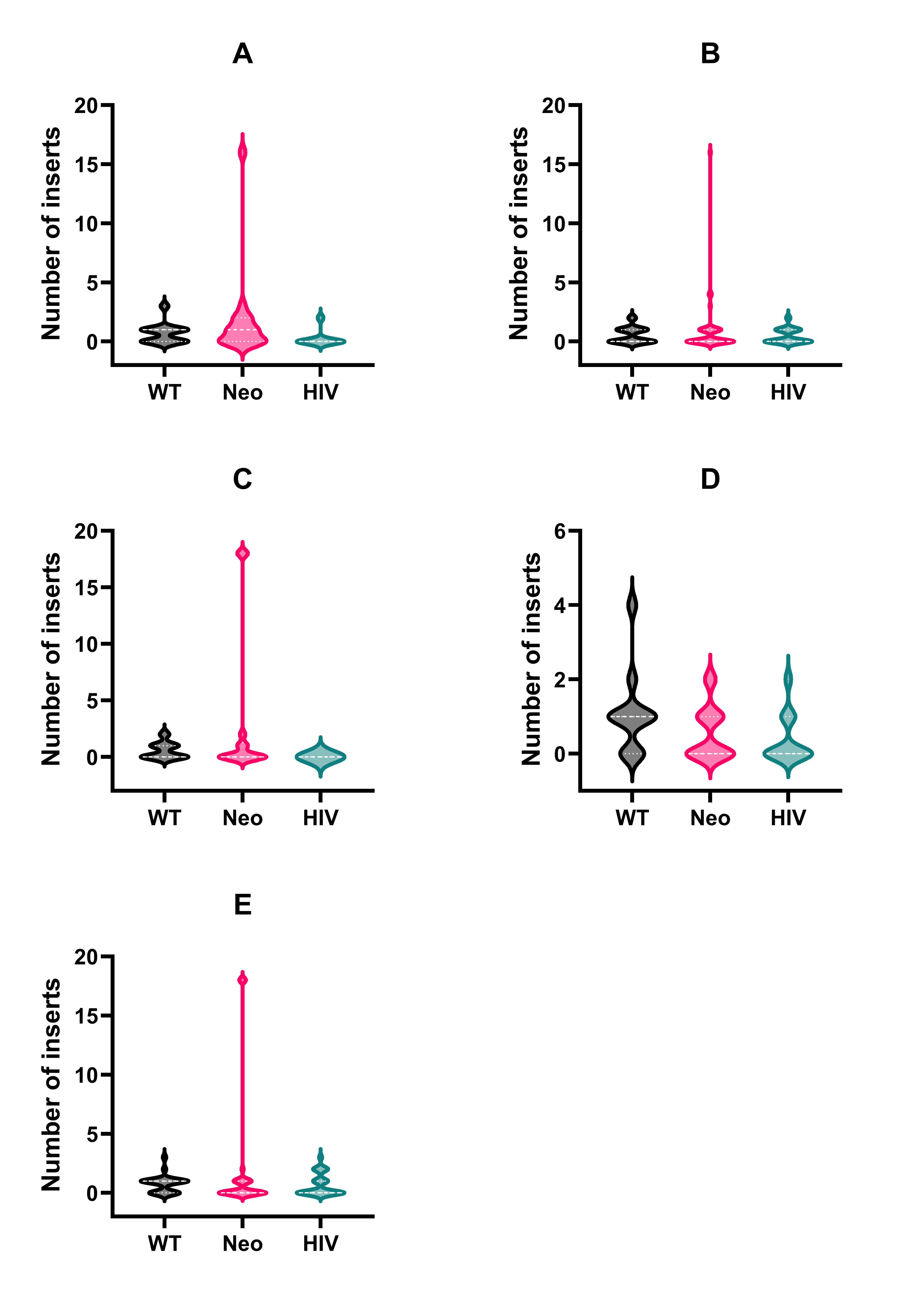

Supplement: Supplementary file 6 — Figure S6. [file CAM4-12-13486-s009.tif]
